# Beyond Competition: Incorporating Positive Interactions between Species to Predict Ecosystem Invasibility

**DOI:** 10.1371/journal.pbio.0060162

**Published:** 2008-06-24

**Authors:** Fabio Bulleri, John F Bruno, Lisandro Benedetti-Cecchi

## Abstract

Incorporating positive species interactions into models relating native species richness to community invasibility will increase our ability to forecast, prevent, and manage future invasions.

One of the many unintended consequences of global commerce has been the translocation of countless plants and animals to new regions, continents, and oceans [1,2]. Such “exotic” species have colonized nearly every habitat on Earth, and modern ecosystems are now made up largely of species originating from geographically distinct regions [3–5]. Most exotic species have negligible or no negative effects, but a small handful have had substantial impacts on native species and ecosystem processes [3,6]. For example, the introduction of the Nile perch (Lates niloticus) into Lake Victoria has not only caused the extinction of two-thirds of the endemic fish fauna, but has changed the entire food web of the lake by reducing the grazing by phytoplanktivores [7,8].

Given the sizable ecological and economic costs of species invasions [9], understanding the environmental factors that regulate them has become a major goal for basic and applied ecologists. One major research theme is the investigation of the relationship between native species richness (the number of local native species) and the ability of exotic species to colonize and thrive in new habitats (termed community “invasibility”) [10,11]. A longstanding concept in ecology is that habitats with high levels of diversity are difficult to invade (the biotic resistance hypothesis—see Glossary) [11–15]. This is because, in theory, a more diverse assemblage of plants or animals can utilize resources more fully than a less diverse community, thus increasing the intensity of competition and making it harder for new species to become established. Predictions from this model are, however, based on the assumption that natural communities are largely structured by competitive interactions and that the effects of native species on invaders are predominantly negative.

**Glossary**

**The biotic resistance hypothesis**, formulated by Elton in 1958 [12], predicts that introduced species often fail to invade communities because strong biotic interactions with native species hinder their establishment and spread. It builds on the assumption that more diverse resident communities, generating more biomass and using resources more completely, would resist the establishment of invaders. Such effects could be due either to **complementarity** in the use of resources among species or to the enhanced probability of including highly competitive species (strong resource users) at high diversity levels (**identity** or **sampling** effect; [13–15]).

**Facilitation** is a biotic interaction in which at least one of the species involved benefits from the presence of the other(s), and neither is negatively affected. Facilitation includes interactions between co-evolved, mutually obligate organisms as well as facultative interactions between species that are not evolutionarily linked. The presence of one species can facilitate another directly, by improving environmental conditions (e.g., reducing stress due to physical and/or chemical conditions), or indirectly, by lessening consumer and/or competition pressure.

**The fluctuating resource hypothesis**, developed by Davis et al. [26], predicts that pronounced fluctuations in resource availability will foster community invasibility. The theory is based on the assumption that an invading species must have access to available resources (e.g., light, nutrients, water for plants) and that a species will have greater success in invading a community if it does not encounter intense competition for these resources from resident species. An increase in resource availability can occur either because the rate at which resources are supplied from external sources is faster than the rate at which the resident assemblage can use them, or because the resident assemblage's use of resources declines.

**Functional traits** are defined as the characteristics of an organism that determine its performance in response to the environment and/or its effects on ecosystem functioning. Variation between individuals or species in traits such as phenology, architecture, resource acquisition, and allocation will influence the success of a population or community. Community structure can be simplified by categorizing species into **functional groups** based on suites of correlated traits.

There is, however, growing evidence that facilitation (positive species interactions—see Glossary) plays an equally important role in shaping communities and ecosystems [16–20]. One species can facilitate another by ameliorating stressful abiotic conditions or by providing refuges from natural enemies such as predators. Nonetheless, positive species interactions are rarely incorporated into conceptual ecological theories that describe the complex dynamics of species invasions [19,21]. Facilitation has been included in invasion scenarios to describe the case of extant exotic species enhancing the colonization of new exotics (e.g., invasional meltdown [22]). Yet a large body of evidence from terrestrial and marine habitats indicates that native species also commonly facilitate exotic colonizers through a variety of mechanisms. For example, shading by the native shrub, Atriplex vesicaria, fosters the establishment of the exotic succulent, Orbea variegata, in South Australia [23], while native sessile invertebrates protect the introduced oyster, Crassostrea gigas, from predation on the rocky shores of Western Canada [24].

## Including Facilitation in Resource-Based Invasion Theory

Incorporating facilitation into ecological theories that can be applied to species invasions could advance our understanding of the processes underlying the colonization and spread of exotic species. For example, our expectations of how species richness and resource availability affect invasibility can be dramatically altered when positive effects of extant species (including natives and established exotics) on exotic invaders are taken into account.

The potential role of facilitation in modifying the diversity–invasibility relationship can be illustrated by constructing a series of simple models that relate species richness to invasibility under different scenarios of community assembly. Two basic assumptions of the models are: (1) that the relationship between species richness and resource availability is negative [25,26] and (2) that the probability that the native assemblage includes facilitators is positively correlated with extant species richness [13,15,19,27,28]. Since invasion success will be greater when an exotic species does not have to compete with residents for resources, any factor causing a temporary increase in resource availability will increase a community's vulnerability to invasion (the fluctuating resource hypothesis—see Glossary) [26]. Near-complete exploitation of resources can, however, occur in both species-rich and species-poor assemblages, so that invasibility is not necessarily related to species richness [26].

**Figure 1:**
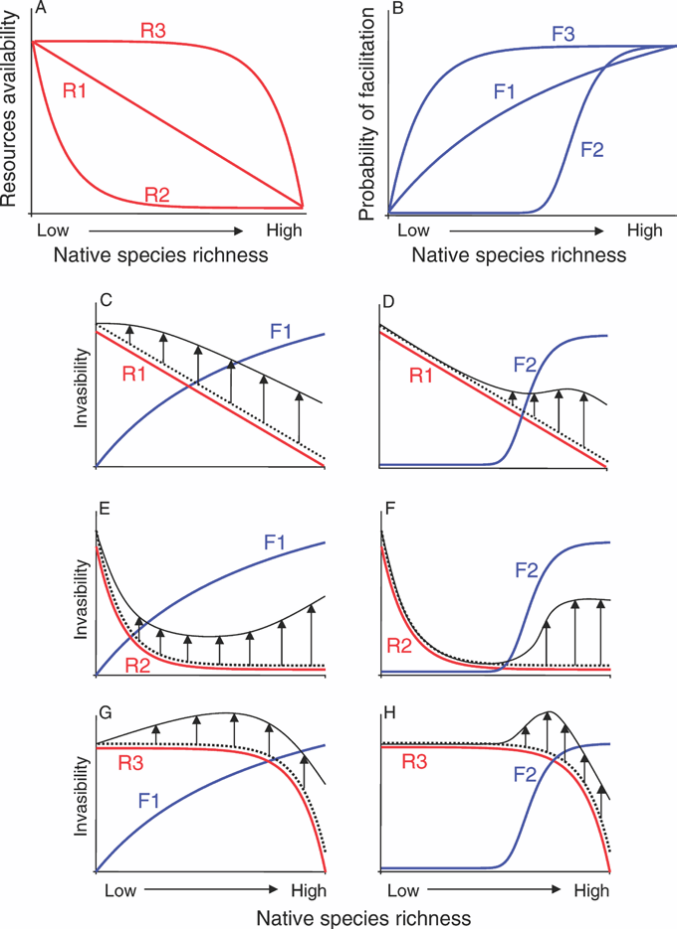
The Interplay between Resource Availability and Facilitation Regulates Invasibility According to both the distribution of HEER and facilitating traits within the native species pool and realistic assembly rules, different curves can be identified to describe how (A) the availability of resources and (B) the probability of exotics species being facilitated by natives vary as functions of native species richness (see text for details). Invasibility varies with native species diversity as a net outcome of two processes: resource depletion (red line) and facilitation (blue line). A combination of R and F curves (C–H) determines the mismatch (upward arrows) between predictions of invasibility as a plain function of resource availability (dotted line) and predictions that account for facilitation (solid black line).

In fact, different modalities of resource depletion (R curves; Figure 1A), as a function of native species richness, can be identified according to (1) the occurrence of functional traits (see Glossary) that confer high efficiency in exploiting resources (HEER); (2) the distribution of HEER traits across the pool of native species; and (3) realistic assembly rules (i.e., the frequency with which a species occurs or its order of appearance/disappearance in disturbed habitats). When HEER traits are not represented in the pool of natives, each new species added (with a constant number of individuals) will use a similar amount of resources, resulting in a linear decay with increasing species richness (R1). For example, Hooper and Vitousek [25] found a linear relationship between plant functional group richness and resource use (nitrogen, phosphorus) when nitrogen-fixers were excluded. When HEER traits are uniformly distributed across the native species pool, a small subset of natives can almost completely deplete resources (R2). The same scenario can take place when HEER traits are not distributed uniformly across native species, but are an exclusive characteristic of common or early successional species. Resources are quickly exploited at low diversity in this scenario. The opposite situation—that is, an almost complete use of resources at high levels of species richness (R3)—occurs when HEER traits are possessed by rare or late successional native species. There are several examples in the ecological literature of both patterns of distribution of HEER traits among species [25,29].

In contrast to resource depletion modalities, the shape of the curves describing the probability of including facilitators in the native assemblage as a function of species richness is yet to be determined empirically. Nonetheless, different curves can be drawn for heuristic purposes according to the distribution of the relevant “facilitating traits,” which ascribe facilitating effects to native species, and to assembly rules (F curves; Figure 1B). If these traits are uniformly distributed across the pool of natives, the probability of including facilitators will follow a linear increase at increasing levels of species richness (F1). For example, on Mediterranean rocky reefs, both encrusting and turf-forming algae facilitate the anchoring of stolons of the exotic alga, Caulerpa racemosa, by providing a more complex substratum than bare rock [30,31]. Greatest invasion success occurs, therefore, at low (encrusting corallines only) to intermediate (encrusting corallines plus algal turfs) levels of native species/functional richness.

Conversely, when facilitating traits are not uniformly distributed across native species and are not possessed by dominant species or early colonizers in disturbed habitats, the probability of including facilitators will be greater at high species richness (F2). There is some empirical evidence for this case as well. For example, the establishment of weeds in California coastal prairies is enhanced by the native nitrogen-fixing shrub, Lupinus arboreus [32]; this shrub is not a dominant component of native communities and is more likely to be part of the native pool when species richness is high.

Finally, when facilitating traits are possessed by few dominant or habitat-forming species, the probability of facilitation will be sustained across the entire range of species richness (F3). Some exotic and native species rely on the presence of species-specific traits within the resident community [33–35]. For example, in the alpine zone of the Chilean Andes, the establishment of the exotic forb, Taraxacum officinale, depends on the presence of cushions formed by the native plant, Azorella monantha [36].

Including positive effects of natives on exotic species drastically modifies predictions based on resource depletion (Figure 1C–1H; only F1 and F2 curves are illustrated for the sake of brevity), since facilitation of exotics by natives can counterbalance the effects of competition. Facilitation can cause invasibility to deviate from the near-universal prediction of a decline with increasing species richness (Figure 1C and 1D). The distribution of facilitating traits across the native species pool determines the species richness level at which invasibility deviates from linearity. Even when resources are monopolized by a small number of species or functional groups, invasibility can be sustained by facilitation (Figure 1E) and, indeed, rise at high species richness (Figure 1F). Also, facilitation can boost invasibility at intermediate (Figure 1G) to high (Figure 1H) levels of species richness, when high native species richness insignificantly reduces the availability of resources.

How does this conceptual model relate to our current understanding of the biodiversity–invasibility relationship? Conflicting results have emerged between small-scale experimental studies, which have typically found a negative relationship between native and exotic species richness [34,37,38], and large-scale observational studies [34,39–41], which have frequently found the opposite in nature. A positive correlation between native and exotic diversity could arise at large spatial scales because the response of both native and exotic species to heterogeneity in abiotic factors at such scales overwhelms the positive effects of diversity on invasion resistance that prevail at smaller scales [27,42,43]. Alternatively, this positive correlation could be due to facilitation of exotics by natives [19,28]. Facilitation is a scale-dependent process, because the larger the area over which the observation/manipulation is conducted, the larger the number of native species (and potential facilitating traits) that are included. Scant experimental evidence for a positive native–exotic species relationship could be, therefore, due to the fact that the spatial scales at which biodiversity manipulations are generally carried out are too small to sample most of the native species/functional traits or to include large-sized species. Alternatively, competition could inherently operate at a smaller spatial scale than facilitation, and thus be more likely to drive the results of small-scale studies.

## From Theory to Practice: Implications for Management

Taking into account positive native–exotic relationships has important implications for intervention strategies targeting biological invasions. Such strategies are based on different approaches: direct eradication of the invader (by means of either biological or mechanical/chemical tools) or deliberate modification of physical and biological features of the receiving system [44–46]. The feasibility of the physical elimination of an invader is independent of the attributes of resident assemblages (although eradication techniques may not be). In contrast, actions commonly prescribed to control invaders by targeting physical and biological features of natural systems, such as the manipulation of disturbance regimes (e.g., fire, grazing, mowing) and nutrient availability or the restoration of native species richness, are commonly grounded in resource-based invasion theory [2,46,47]. They do not take into account the dual nature of species interactions, and might yield unanticipated surprises. For example, the use of ecosystem engineers [48] (e.g., species that create or modify habitats) or facilitation in general [49] in restoration practises, while enhancing the recovery of targeted native species and, likely, overall biodiversity, could unintentionally create new opportunities for invaders [50]. This could be the case with the native eelgrass, Zostera marina, which enhances the establishment of Sargassum muticum on soft-sediments by trapping drifting fragments of the invasive macroalga [51]. Hence, the restoration of seagrass meadows, while benefiting a large number of native species [52], could also foster invasion.

Considering positive interactions among species within the same trophic level can clearly alter our expectations of the role of native species richness in determining the success of exotic plants and animals. The next step will be to incorporate multiple trophic levels and consumptive interactions. Taking into account herbivory and/or predation would generate a multidimensional model; this could likely occur as our understanding of the mechanisms regulating interactions among species at different trophic levels advances. The effects of resource availability and facilitation on the ability of an exotic species to become established within a recipient community would be, in fact, modified by the outcome of a complex web of direct and indirect interactions, varying in direction and strength. But given the prominent role of facilitation in mitigating consumer effects on prey populations, a broad view would likely bolster the realized net role of positive interactions and further modify diversity–invasibility relationships.

Predictions of future invasion scenarios and management strategies based on a single side of the coin (negative interactions) will yield limited predictive power and problem-solving capability. A unified theory of invasibility must, therefore, include the attributes of both native and invading species, enabling the assessment of the counterbalance between positive and negative interactions.

## Abbreviations

HEER: high efficiency in exploiting resources
